# Supplementary material for: Covalent-supramolecular hybrid polymers as muscle-inspired anisotropic actuators
Source: Nat Commun. 2018 Jun 19;9:2395. doi: 10.1038/s41467-018-04800-w (PMC6008453; doi:10.1038/s41467-018-04800-w)
Supplement: Supplementary file 1 — Supplementary Information [file 41467_2018_4800_MOESM1_ESM.pdf]

Supplementary Information for

Covalent-supramolecular hybrid polymers as muscle-inspired anisotropic actuators

Chin *et al.*

Supplementary Note 1 | NMR peak assignments.

PA1 ^{13}C-NMR (125 MHz, TFA-d_1): δ 13.5 (Pal-$\underline{C}H_3$), 16.8 – 17.1 (3x Ala-$\underline{C}_{(\beta)}H_3$), 17.9 (2x Val-$\underline{C}_{(\gamma)}H_3$), 18.2 (Val-$\underline{C}_{(\gamma)}H_3$), 18.6 (Val-$\underline{C}_{(\gamma)}H_3$), 18.7 (Val-$\underline{C}_{(\gamma)}H_3$), 18.8 (Val-$\underline{C}_{(\gamma)}H_3$), 23.2 (several Pal-$\underline{C}H_2$), 23.4 (Lys-$\underline{C}_{(\gamma)}H_2$), 27.1 (Pal-$\underline{C}_{(\beta)}H_2$), 27.5 (2x Glu-$\underline{C}_{(\beta)}H_2$), 27.6 (Glu-$\underline{C}_{(\beta)}H_2$), 28.8 (Lys-$\underline{C}_{(\delta)}H_2$), 29.7 – 30.4 (3x Glu-$\underline{C}_{(\gamma)}H_2$, several Pal-$\underline{C}H_2$), 31.8 (2x Bib-$\underline{C}H_3$), 31.9 (Val-$\underline{C}_{(\beta)}H$), 32.0 (Val-$\underline{C}_{(\beta)}H$), 32.1 (Lys-$\underline{C}_{(\beta)}H_2$), 32.3 (Val-$\underline{C}_{(\beta)}H$), 32.7 (several Pal-$\underline{C}H_2$), 35.7 (Pal-$\underline{C}_{(\alpha)}H$), 41.3 (Lys-$\underline{C}_{(\varepsilon)}H_2$), 51.1 (2x Ala-$\underline{C}_{(\alpha)}H$), 51.4 (Ala-$\underline{C}_{(\alpha)}H$), 54.4 (2x Glu-$\underline{C}_{(\alpha)}H$), 54.6 (Glu-$\underline{C}_{(\alpha)}H$), 55.1 (Lys-$\underline{C}_{(\alpha)}H$), 60.4 ($\underline{C}(CH_3)_2Br$), 60.9 (Val-$\underline{C}_{(\alpha)}H$), 61.5 (Val-$\underline{C}_{(\alpha)}H$), 62.1 (Val-$\underline{C}_{(\alpha)}H$), 173.6 (Val-$\underline{C}O$), 174.2 ($\underline{C}O$), 174.3 ($\underline{C}O$), 174.5 ($\underline{C}O$), 174.7 ($\underline{C}O$), 175.5 ($\underline{C}O$), 175.8 ($\underline{C}O$), 176.0 ($\underline{C}O$), 176.4 ($\underline{C}O$), 177.5 (Bib-$\underline{C}O$), 178.8 (Lys-$\underline{C}O$), 180.5 (Glu-$\underline{C}_{(\delta)}O$), 180.6 (2x Glu-$\underline{C}_{(\delta)}O$), 181.6 (Pal-$\underline{C}O$);

^{1}H-NMR (600 MHz, TFA-d_1): δ 0.80 (t, 3H, J = 6.5 Hz, Pal-C$\underline{H}_3$), 0.92 – 0.99 (m, 18H, 6x Val-$C_{(\gamma)}\underline{H}_3$), 1.21 – 1.37 (m, 24H, several Pal-C$\underline{H}_2$), 1.44 – 1.54 (m, 11H, 3x Ala-$C_{(\beta)}\underline{H}_3$, Lys-$C_{(\gamma)}\underline{H}_2$), 1.63 – 1.73 (m, 4H, Lys-$C_{(\delta)}\underline{H}_2$, Pal-$C_{(\beta)}\underline{H}_2$), 1.84 – 1.99 (m, 8H, 2x Bib-C$\underline{H}_3$, Lys-$C_{(\beta)}\underline{H}_2$), 2.04 – 2.21 (m, 5H, Glu-$C_{(\beta)}\underline{H}_2$, 3x Val-$C_{(\beta)}\underline{H}$), 2.25 – 2.34 (m, 4H, 2x Glu-$C_{(\beta)}\underline{H}_2$), 2.58 – 2.64 (m, 8H, 3x Glu-$C_{(\gamma)}\underline{H}_2$, Pal-$C_{(\alpha)}\underline{H}_2$), 3.36 (t, 2H, J = 6.60 Hz, Lys-$C_{(\varepsilon)}\underline{H}_2$), 4.38 (d, 1H, J = 8.24 Hz, Val-$C_{(\alpha)}\underline{H}$), 4.43 (d, 1H, J = 7.85 Hz, Val-$C_{(\alpha)}\underline{H}$), 4.52 (d, 1H, J = 7.73 Hz, Val-$C_{(\alpha)}\underline{H}$), 4.59 – 4.65 (m, 4H, 3x Ala-$C_{(\alpha)}\underline{H}$, Lys-$C_{(\alpha)}\underline{H}$), 4.77 – 4.82 (m, 3H, 3x Glu-$C_{(\alpha)}\underline{H}$);

PA2 ^{13}C-NMR (125 MHz, TFA-d_1): δ 13.5 (Pal-$\underline{C}H_3$), 16.8 (Ala-$\underline{C}_{(\beta)}H_3$), 16.9 (Ala-$\underline{C}_{(\beta)}H_3$), 17.1 (Ala-$\underline{C}_{(\beta)}H_3$), 17.9 (2x Val-$\underline{C}_{(\gamma)}H_3$), 18.2 (Val-$\underline{C}_{(\gamma)}H_3$), 18.6 (Val-$\underline{C}_{(\gamma)}H_3$), 18.7 (Val-$\underline{C}_{(\gamma)}H_3$), 18.8 (Val-$\underline{C}_{(\gamma)}H_3$), 23.2 (several Pal-$\underline{C}H_2$), 27.1 (Pal-$\underline{C}_{(\beta)}H_2$), 27.3 (Glu-$\underline{C}_{(\beta)}H_2$), 27.6 (Glu-$\underline{C}_{(\beta)}H_2$), 27.7 (Glu-$\underline{C}_{(\beta)}H_2$), 29.7 – 30.4 (3x Glu-$\underline{C}_{(\gamma)}H_2$, several Pal-$\underline{C}H_2$), 31.9 (Val-$\underline{C}_{(\beta)}H$), 32.0 (Val-$\underline{C}_{(\beta)}H$), 32.3 (Val-$\underline{C}_{(\beta)}H$), 32.7 (several Pal-$\underline{C}H_2$), 35.7 (Pal-$\underline{C}_{(\alpha)}H_2$), 51.1 ($Ala_{(\alpha)}$-$\underline{C}H$), 51.2 ($Ala_{(\alpha)}$-$\underline{C}H$), 51.4 ($Ala_{(\alpha)}$-$\underline{C}H$), 53.9 ($Glu_{(\alpha)}$-$\underline{C}H$), 54.4 ($Glu_{(\alpha)}$-$\underline{C}H$), 54.7 ($Glu_{(\alpha)}$-$\underline{C}H$), 60.9 ($Val_{(\alpha)}$-$\underline{C}H$), 61.6 ($Val_{(\alpha)}$-$\underline{C}H$), 62.2 ($Val_{(\alpha)}$-$\underline{C}H$), 173.6 (Val-$\underline{C}O$), 174.3 ($\underline{C}O$), 174.4 ($\underline{C}O$), 174.5 ($\underline{C}O$), 174.7 ($\underline{C}O$), 175.5 (Ala-$\underline{C}O$), 176.0 (Ala-$\underline{C}O$), 176.5 (Ala-$\underline{C}O$), 177.7 (Glu-$\underline{C}O$), 180.6 (Glu-$\underline{C}_{(\delta)}O$), 180.6 (Glu-$\underline{C}_{(\delta)}O$), 180.7 (Glu-$\underline{C}_{(\delta)}O$), 181.6 (Pal-$\underline{C}O$);

^{1}H-NMR (600 MHz, TFA-d_1): δ 0.81 (t, 3H, J = 6.6 Hz, Pal-C$\underline{H}_3$), 0.93 – 1.00 (m, 18H, 6x Val-$C_{(\gamma)}\underline{H}_3$), 1.20 – 1.37 (m, 24H, 12x Pal-C$\underline{H}_2$), 1.44 – 1.47 (m, 9H, 3x Ala-$C_{(\beta)}\underline{H}_3$), 1.70 – 1.75 (m, 2H, Pal-$C_{(\beta)}\underline{H}_2$), 2.06 – 2.19 (m, 5H, Glu-$C_{(\beta)}\underline{H}_2$, 3x Val-$C_{(\beta)}\underline{H}$), 2.27 – 2.37 (m, 4H, 2x Glu-$C_{(\beta)}\underline{H}_2$), 2.59 – 2.66 (m, 8H, 3x Glu-$C_{(\gamma)}\underline{H}_2$, Pal-$C_{(\alpha)}\underline{H}_2$), 4.39 (d, 1H, J = 8.4 Hz, Val-$C_{(\alpha)}\underline{H}$), 4.44 (d, 1H, J = 8.0 Hz, Val-$C_{(\alpha)}\underline{H}$), 4.53 (d, 1H, J = 7.7 Hz, Val-$C_{(\alpha)}\underline{H}$), 4.60 – 4.66 (m, 3H, 3x Ala-$C_{(\alpha)}\underline{H}$), 4.78 – 4.85 (m, 3H, 3x Glu-$C_{(\alpha)}\underline{H}$);

Supplementary Note 2 | Purity of peptide amphiphile materials. The purity of the PA materials used in this work was determined to be >95% from integrating the absorption signals at 220 nm (**Supplementary Figure 1**, left column). The two peaks in the analytical LC trace of **PA3** (**Supplementary Figure 1c**) at an elution time of 16 and 16.3 minutes correspond to two isomers of the TAMRA dye. The mass spectra shown in the right column of **Supplementary Figure 1** were obtained from the whole peak region of the respective LC trace. **Supplementary Tables 1-3** list the species with a minimum ion count signal of 2% of the highest intensity in the spectrum. The majority of the identified signals correspond to the expected masses of **PA1-3** or their clusters, with proton-, sodium-, or ammonia adducts or combinations thereof. In the case of **PA1** and **PA2**, a trace amount of contamination was observed with a species that had acquired an additional alanine, termed as **PA4** and **PA5**, respectively. The addition of an amino acid such as alanine is a common occurrence in solid phase peptide synthesis and such impurities are very difficult to completely remove through HPLC purification. In our experience, the self-assembled structures of **PA1** and **PA2** were not affected by the presence of trace amounts of **PA4** and **PA5**.

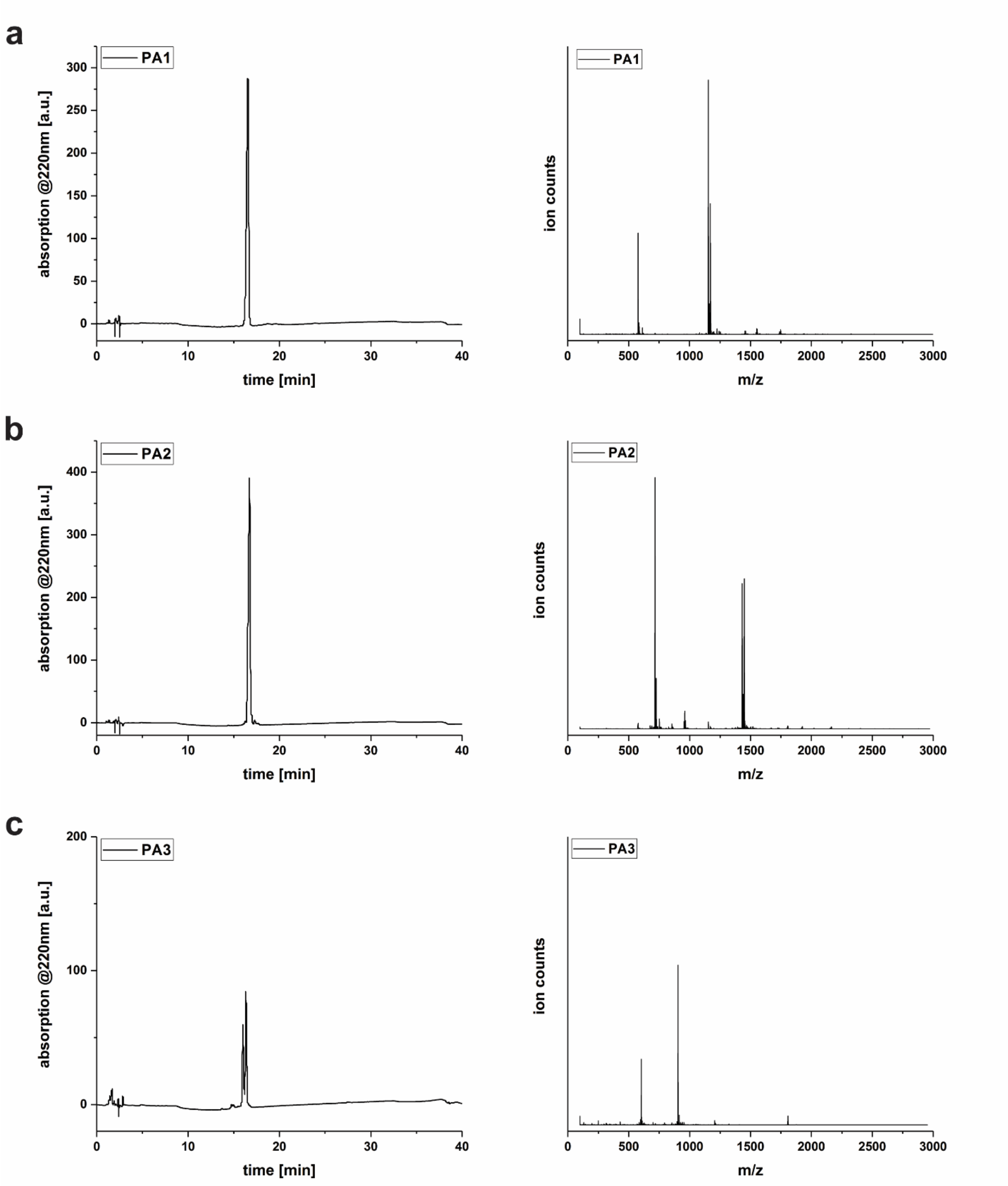

Supplementary Figure 1 | LC-MS traces (left) of (**a**) **PA1**, (**b**) **PA2**, and (**c**) **PA3** from purity analysis. The mass spectra (right) are obtained by integrating the ion counts from the full peak region in the LC-MS trace.

Supplementary Table 1 | Ion assignments, chemical formula, theoretical m/z values, and deviation between experimental and theoretical values of signals in the mass spectrum of HPLC purified PA sample obtained from LC-MS (see **Supplementary Figure 1a**). All signals with an intensity > 2% of the most intense signal in the spectrum are listed here. The structure **PA4** is that of **PA1**, but with an additional alanine in the sequence, which is a common occurrence in solid phase peptide synthesis.

$m/z_{expt.}$	Ion Assignment	Formula	$m/z_{theor.}$	Δ m/z
577.2859	[**PA1** + 2 H]$^{2+}$	$[C_{55}H_{98}N_{10}O_{16}]^{2+}$	577.3576	0.0717
585.7971	[**PA1** + NH_4 + H]$^{2+}$	$[C_{55}H_{101}N_{11}O_{16}]^{2+}$	585.8708	0.0737
612.8002	[**PA4** + 2 H]$^{2+}$	$[C_{58}H_{103}N_{11}O_{17}]^{2+}$	612.8761	0.0759
1153.5993	[**PA1** + H]$^{+}$	$[C_{55}H_{97}N_{10}O_{16}]^{+}$	1153.7079	0.1086
1159.2657	[3 **PA1** + NH_4 + 2H]$^{3+}$	$[C_{165}H_{294}N_{31}O_{48}]^{3+}$	1159.3834	0.1177
1162.1066	[2 **PA1** + NH_4 + H]$^{2+}$	$[C_{110}H_{197}N_{21}O_{32}]^{2+}$	1162.2210	0.1144
1164.5879	[2 **PA1** + Na + H]$^{2+}$	$[C_{110}H_{193}N_{20}NaO_{32}]^{2+}$	1164.6988	0.1109
1170.6220	[**PA1** + NH_4]$^{+}$	$[C_{55}H_{100}N_{11}O_{16}]^{+}$	1170.7344	0.1124
1175.5756	[**PA1** + Na]$^{+}$	$[C_{55}H_{96}N_{10}NaO_{16}]^{+}$	1175.6898	0.1142
1224.6253	[**PA4** + H]$^{+}$	$[C_{58}H_{102}N_{11}O_{17}]^{+}$	1224.7450	0.1197
1549.1489	[4 **PA1** + 2 NH_4 + H]$^{3+}$	$[C_{220}H_{393}N_{42}O_{64}]^{3+}$	1549.2924	0.1435
1554.8224	[4 **PA1** + 3 NH_4]$^{3+}$	$[C_{220}H_{393}N_{43}O_{64}]^{3+}$	1554.9679	0.1455

Supplementary Table 2 | Ion assignments, chemical formula, theoretical m/z values, and deviation between experimental and theoretical values of signals in the mass spectrum of HPLC purified PA sample obtained from LC-MS (see **Supplementary Figure 1b**). All signals with an intensity > 2% of the most intense signal in the spectrum are listed here. The structure **PA5** is that of **PA2**, but with an additional alanine in the sequence, which is a common occurrence in solid phase peptide synthesis.

$m/z_{expt.}$	Ion Assignment	Formula	$m/z_{theor.}$	Δ (m/z)
577.2850	[**PA1** + 2 H]$^{2+}$	$[C_{55}H_{98}N_{10}O_{16}]^{2+}$	577.3576	0.0726
715.3001	[**PA2** + 2H]$^{2+}$	$[C_{65}H_{115}BrN_{12}O_{18}]^{2+}$	715.3812	0.0811
723.8114	[**PA2** + NH_4 + H]$^{2+}$	$[C_{65}H_{118}BrN_{13}O_{18}]^{2+}$	723.8945	0.0831
750.8145	[**PA5** + 2 H]$^{2+}$	$[C_{68}H_{120}BrN_{13}O_{19}]^{2+}$	750.8998	0.0853
853.3498	unknown double charged	-	-	-
953.4043	[2 **PA2** + 3 H]$^{3+}$	$[C_{130}H_{229}Br_2N_{24}O_{36}]^{3+}$	953.5059	0.1016
959.0796	[2 **PA2** + NH_4 + 2 H]$^{3+}$	$[C_{130}H_{232}Br_2N_{25}O_{36}]^{3+}$	959.1814	0.1018
964.7538	[2 **PA2** + 2 NH_4 + H]$^{3+}$	$[C_{130}H_{235}Br_2N_{26}O_{36}]^{3+}$	964.8569	0.1031
1153.5943	[**PA1** + H]$^{+}$	$[C_{55}H_{97}N_{10}O_{16}]^{+}$	1153.7079	0.1136
1429.6266	[**PA2** + H]$^{+}$	$[C_{65}H_{114}BrN_{12}O_{18}]^{+}$	1429.7552	0.1286
1435.2900	[3 **PA2** + NH_4 + 2 H]$^{3+}$	$[C_{195}H_{345}Br_3N_{37}O_{54}]^{3+}$	1435.4307	0.1407
1437.6231	unknown double charged	-	-	-
1442.6340	unknown triple charged	-	-	-
1446.6519	[**PA2** + NH_4]$^{+}$	$[C_{65}H_{117}BrN_{13}O_{18}]^{+}$	1446.7817	0.1298

Supplementary Table 3 | Ion assignments, chemical formula, theoretical m/z values, and deviation between experimental and theoretical values of signals in the mass spectrum of HPLC purified PA sample obtained from LC-MS (see **Supplementary Figure 1c**). All signals with an intensity > 2% of the most intense signal in the spectrum are listed here.

$m/z_{expt.}$	Ion Assignment	Formula	$m/z_{theor.}$	Δ (m/z)
597.3403	unknown triple charged	-	-	-
603.0170	[**PA3** + 3 H]$^{3+}$	$[C_{92}H_{142}N_{15}O_{22}]^{3+}$	603.0146	0.0024
610.3430	[**PA3** + Na + 2 H]$^{3+}$	$[C_{92}H_{141}N_{15}NaO_{22}]^{3+}$	610.3419	0.0011
897.0110	unknown double charged	-	-	-
904.0224	[**PA3** + 2 H]$^{2+}$	$[C_{92}H_{141}N_{15}O_{22}]^{2+}$	904.0183	0.0041
915.0104	[**PA3** + Na + H]$^{2+}$	$[C_{92}H_{140}N_{15}NaO_{22}]^{2+}$	915.0092	0.0012
1205.0218	[2 **PA3** + 3 H]$^{3+}$	$[C_{184}H_{281}N_{30}O_{44}]^{3+}$	1205.0219	0.0001
1807.0271	[**PA3** + H]$^{+}$	$[C_{92}H_{140}N_{15}O_{22}]^{+}$	1807.0292	0.0021

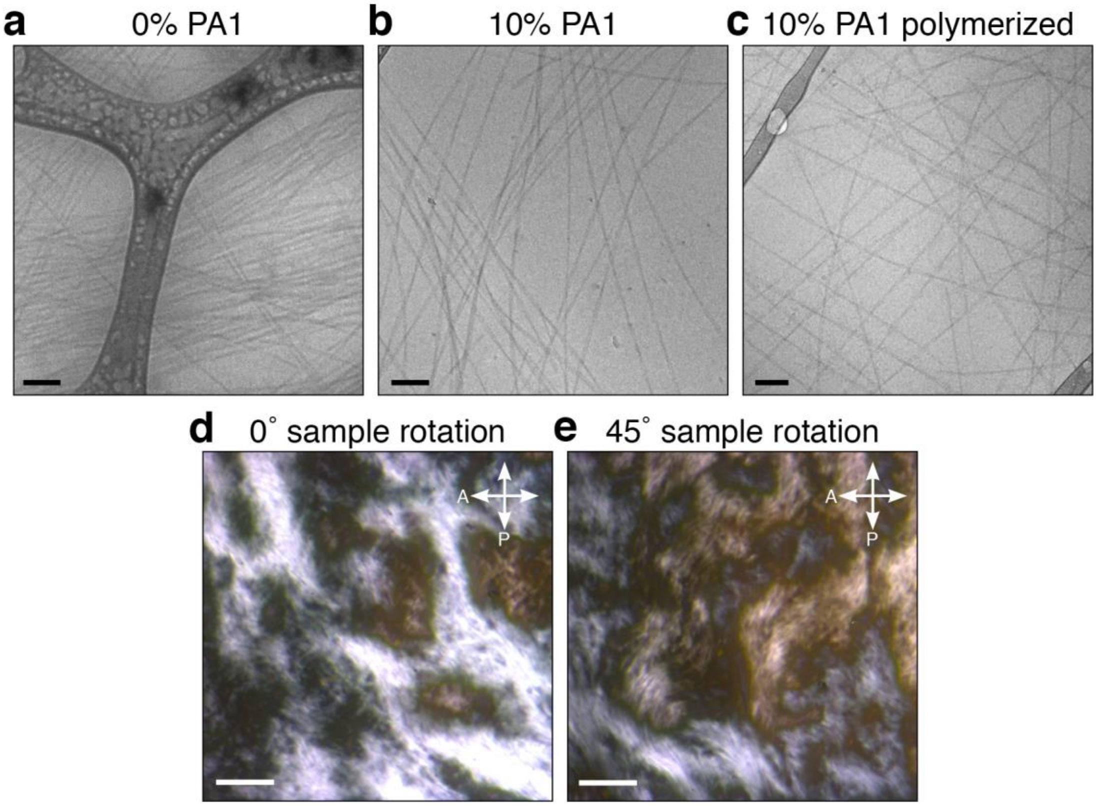

Supplementary Figure 2 | Microscopy of PA nanofiber coassemblies. Cryo-TEM of (**a**), 0 mol% **PA1** + 100 mol% **PA2**; (**b**) 10 mol% **PA1** + 90 mol% **PA2**; (**c**) 10 mol% **PA1** + 90 mol% **PA2** after polymerization of thermoresponsive covalent polymer. Note that due to the similar density of the OEGMA copolymer to water, there is no distinct contrast from water within the polymer phase. (Scale bars are 100 nm). Polarized optical microscopy showing birefringence of a 1 wt% solution of 10 mol% **PA1** + 90 mol% **PA2** (same as panel **b**) after annealing; (**d**) 0˚ sample rotation, (**e**) 45˚ sample rotation. (Scale bars are 200 µm)

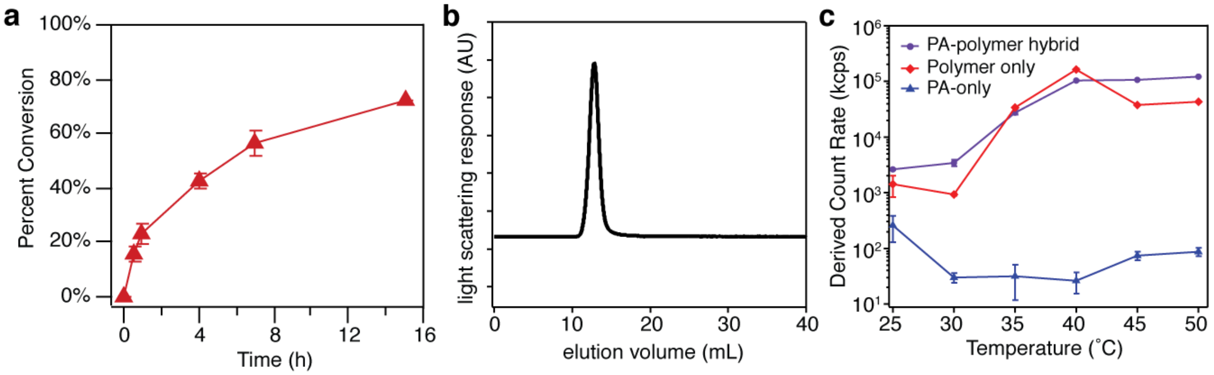

Supplementary Figure 3 | Characterization of covalent phase (**a**) Polymer conversion is monitored by following the decrease in signal intensity of vinyl peaks by ^{1}H NMR. (**b**) Representative GPC In THF of polymer after 15 hours shows a monomodal peak on light scattering detector. M_n = 9.8x10^{4}, PDI = 1.36. (**c**) Light-scattering behavior at increasing temperature, showing increased scattering above LCST for samples containing PEGMA polymers. Error bars represent one standard deviation.

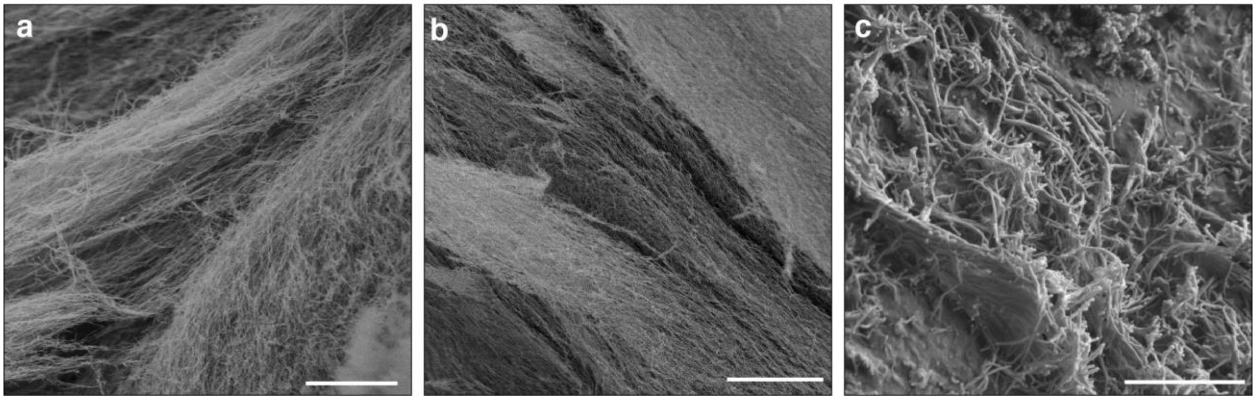

Supplementary Figure 4 | SEM of PA nanofibers in circumferentially aligned tubular hydrogels maintaining alignment (**a**) after gelation with 0.1M $CaCl_2$ solution (scale bar is 5 µm) and (**b**) after polymerization and dialysis (scale bar is 10 µm). (**c**) SEM image of polymer-only hydrogel (scale bar is 2 µm). Note that the polymer hydrogel also has a fibrous structure on small length scales, but does not contain long-range order. Images were taken from within a cross-section of the tube wall.

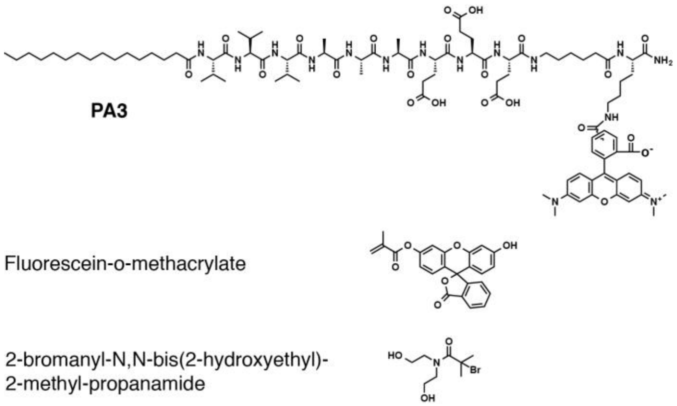

Supplementary Figure 5 | Chemical structures of additional molecules used in characterization and control experiments.

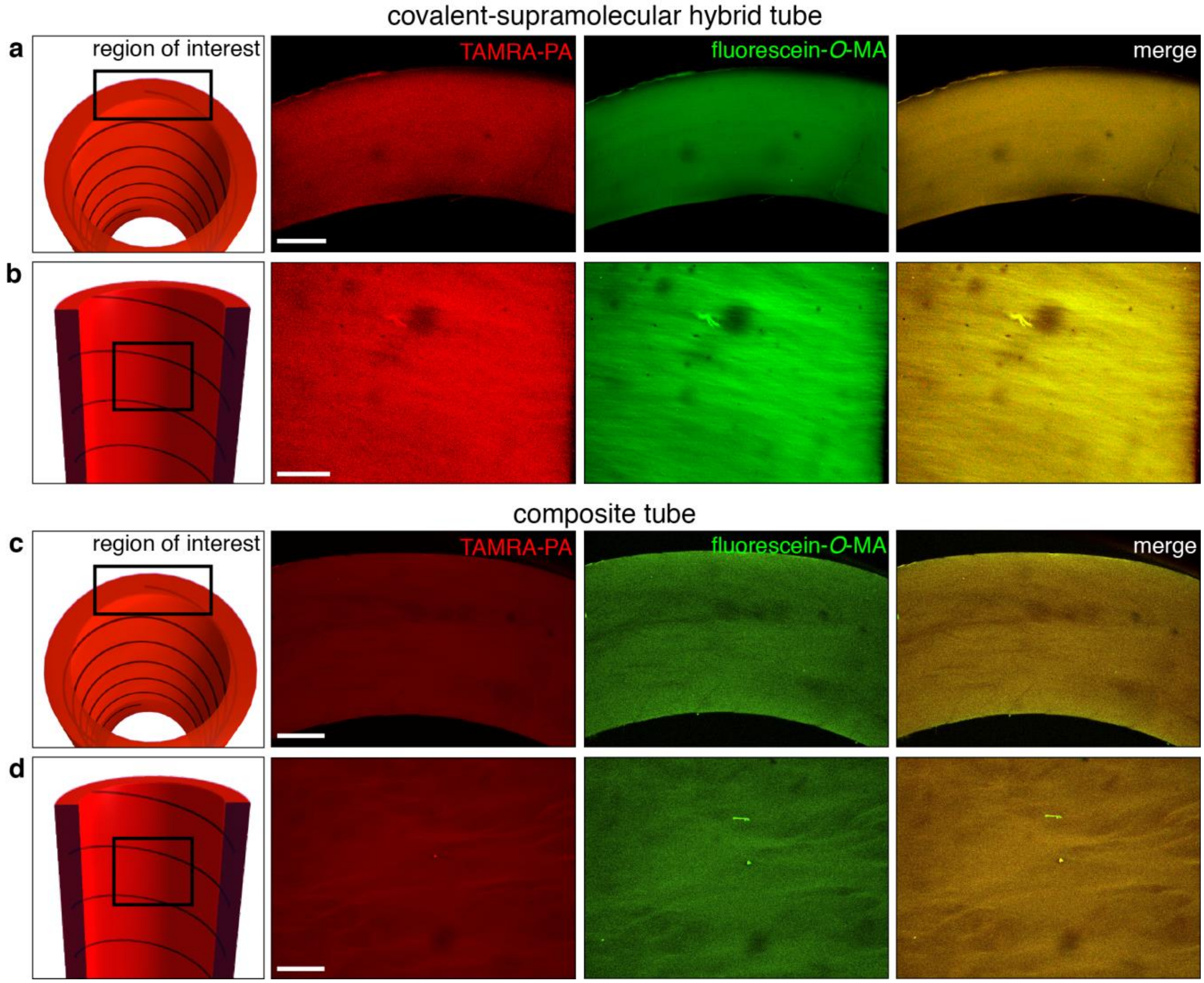

Supplementary Figure 6 | (**a**) Confocal microscopy of fluorescently tagged circumferentially aligned hybrid tube in tube cross-section. (**b**) Confocal microscopy of fluorescently tagged circumferentially aligned hybrid along tube wall. (**c**) Confocal microscopy of fluorescently tagged composite tube in tube cross-section. (**d**) Confocal microscopy of fluorescently tagged composite along tube wall. Fluorescently tagged hybrids and composites contain 1 mol% of **PA3** in the supramolecular phase and 1 monomer wt% of fluorescein-*O*-methacrylate in the covalent phase. Scale bars are 200 μm.

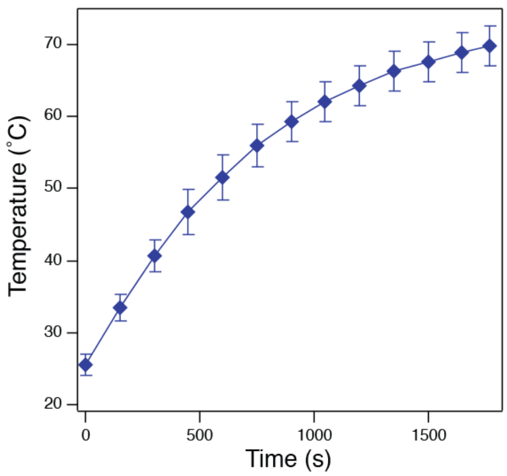

Supplementary Figure 7 | Average heating curve of samples heated in **Fig. 2** and **Fig. 4b**. (mean ± s.d., n = 20)

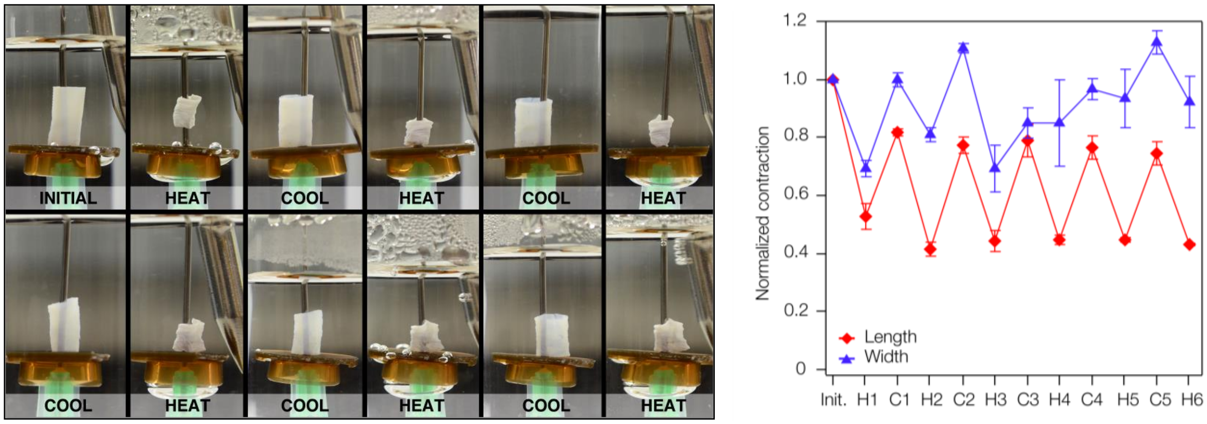

Supplementary Figure 8 | Circumferentially aligned PA-polymer hybrid is able to retain shape and ability to contract across six heating-cooling cycles (data represented as mean ± s.d. of 3 measurements). After repeated handling, width contraction of the hybrids tends to become somewhat ellipsoidal (for example, from touching the central post in the contraction chamber), causing deviations in tube measurement.

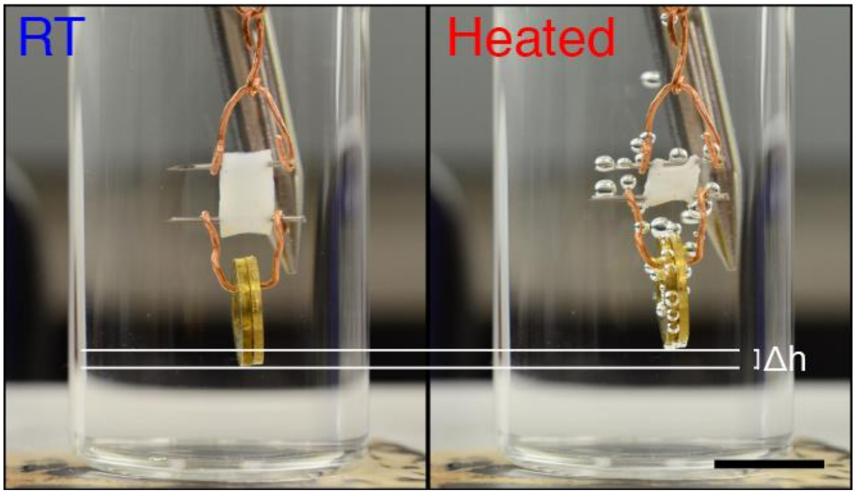

Supplementary Figure 9 | PA-polymer hybrid is able to perform work upon heating. 760 mg weights were attached to a hybrid material (2.0 mg dry weight) and lifted 1.67 mm against gravity (scale bar is 1 cm). This corresponds to a work capacity of 0.629 kJ kg^{-1} and volumetric energy density of 5.656 kJ m^{-3}.

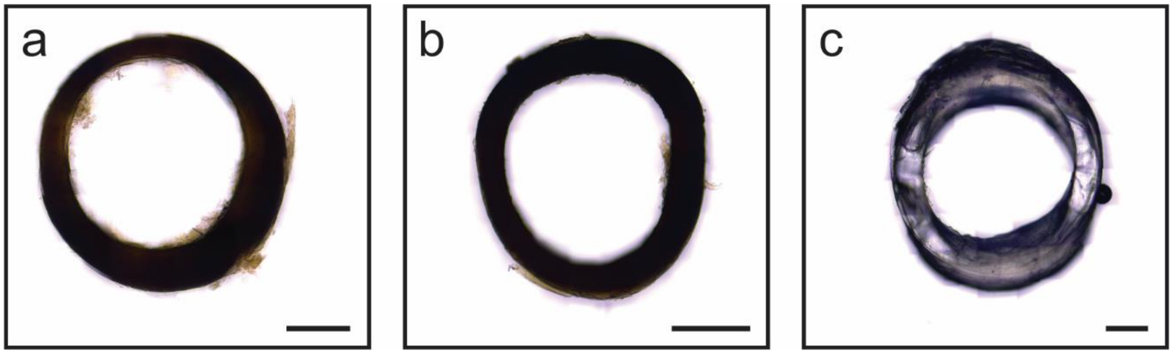

Supplementary Figure 10 | Bright-field microscopy images of cross-sectional samples from (**a**) circumferentially aligned, (**b**) axially aligned, and (**c**) covalent polymer tubular hydrogels, showing the same field of view as the images in **Fig. 3a-c**. Images were stitched using ImageJ plugins. Scale bars are 1 mm.

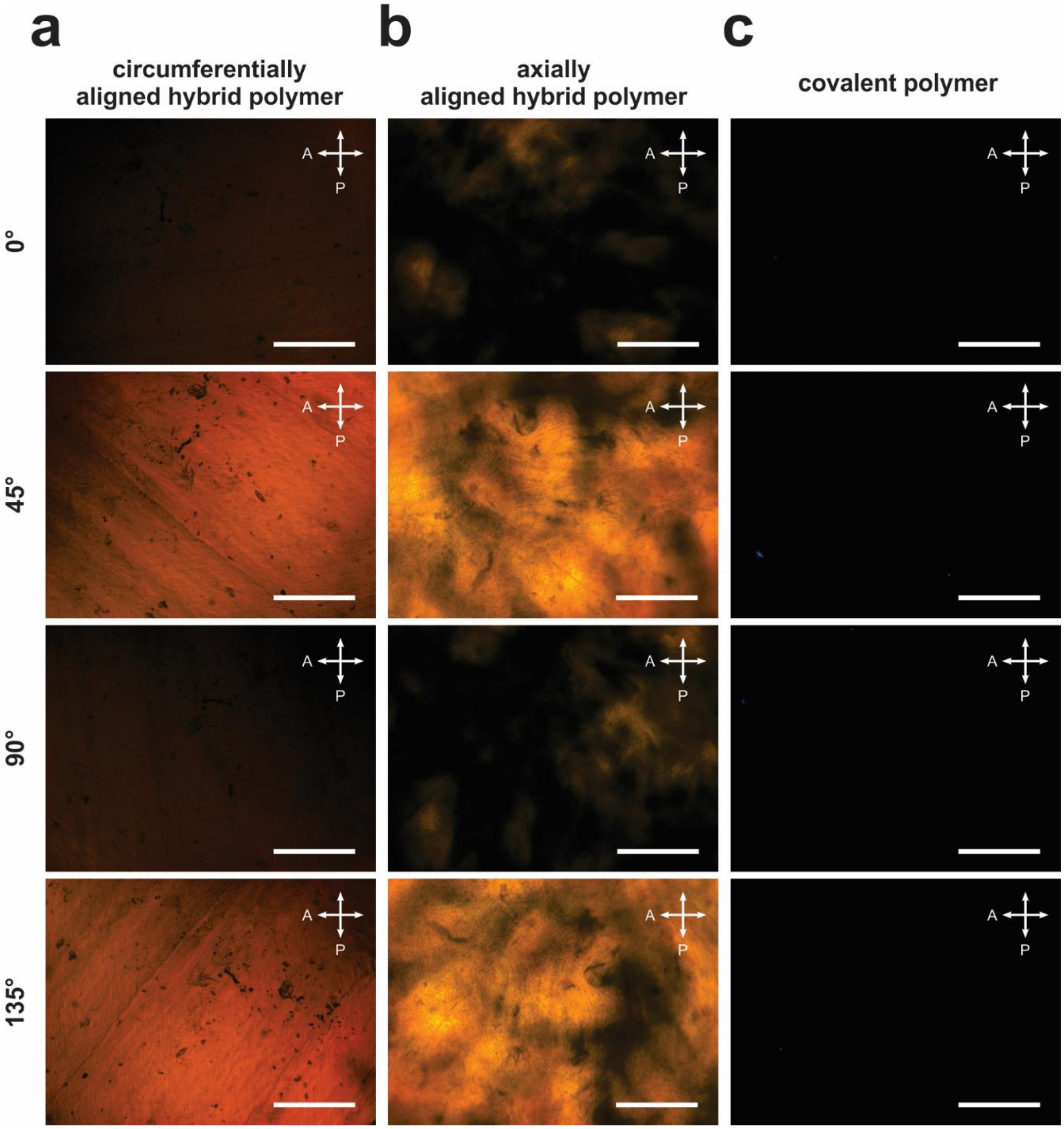

Supplementary Figure 11 | Microscopy images of sections of the tube wall of tubular hydrogels from (**a**) circumferentially aligned hybrid, (**b**) axially aligned hybrid, and (**c**) covalent polymer under cross-polarized light. A sample stage was used to rotate the samples by 0, 45, 90, and 135 degrees, respectively. As expected, the highly circumferentially aligned tube shows strong birefringence at 45 and 135 degrees of sample rotation and minimal birefringence at 0 and 90 degrees. The axially aligned hybrid exhibits the same trend as the circumferentially aligned hybrid, however, domains with different alignment are present. In contrast, the covalent polymer tube shows no birefringence at any sample orientation. The corresponding bright-field images are depicted in **Supplementary Figure 12**. Scale bars are 400 μm.

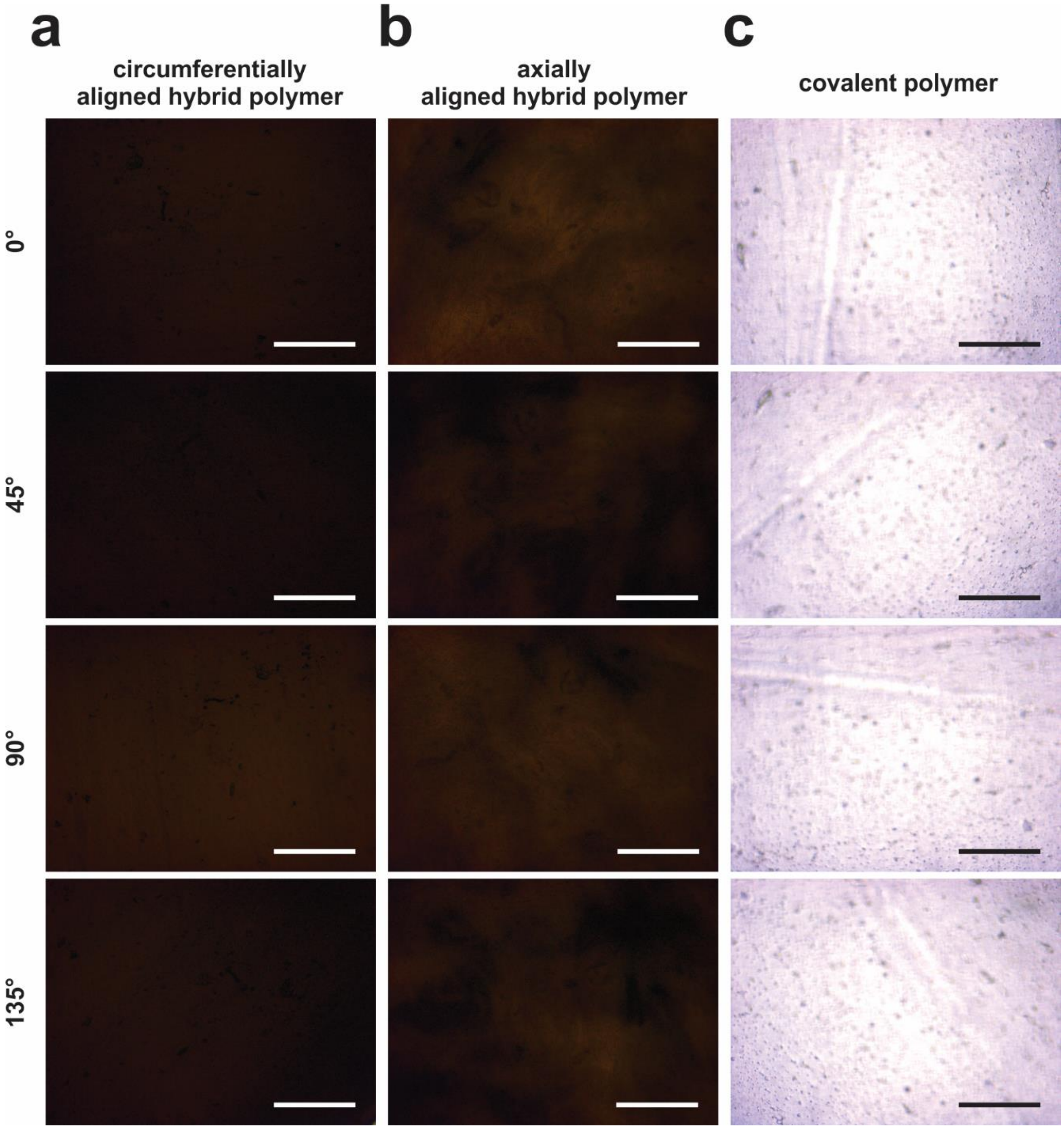

Supplementary Figure 12 | Bright-field microscopy images of the tube wall from (**a**) circumferentially aligned, (**b**) axially aligned, and (**c**) covalent polymer tubular hydrogels corresponding to the same positions as shown in **Supplementary Figure 11**. A sample stage was used to rotate the samples to 0, 45, 90, and 135 degrees, respectively. Scale bars are 400 μm.

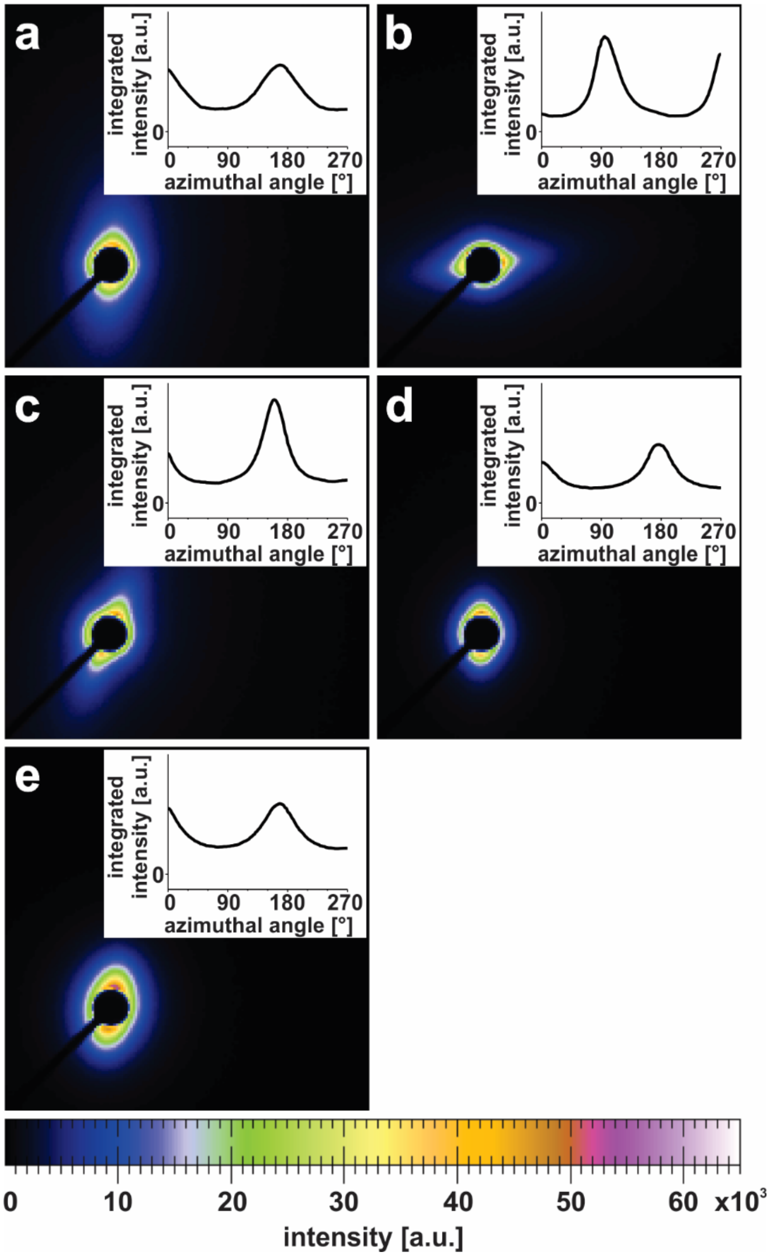

Supplementary Figure 13 | 2D small angle x-ray scattering patterns show angle dependent intensity maxima in the tubular hydrogels. Insets show integrated radial intensity versus azimuthal angle. (**a**) circumferentially aligned and (**b**) axially aligned PA tubes before grafting of covalent polymer. Composite tubular hydrogel (**c**) before and (**d**) after grafting of covalent polymer. **e**) The alignment of the PA nanostructures is maintained within the circumferentially aligned hybrid structures after multiple contraction and expansion cycles (n > 3). All measurements were taken at room temperature.

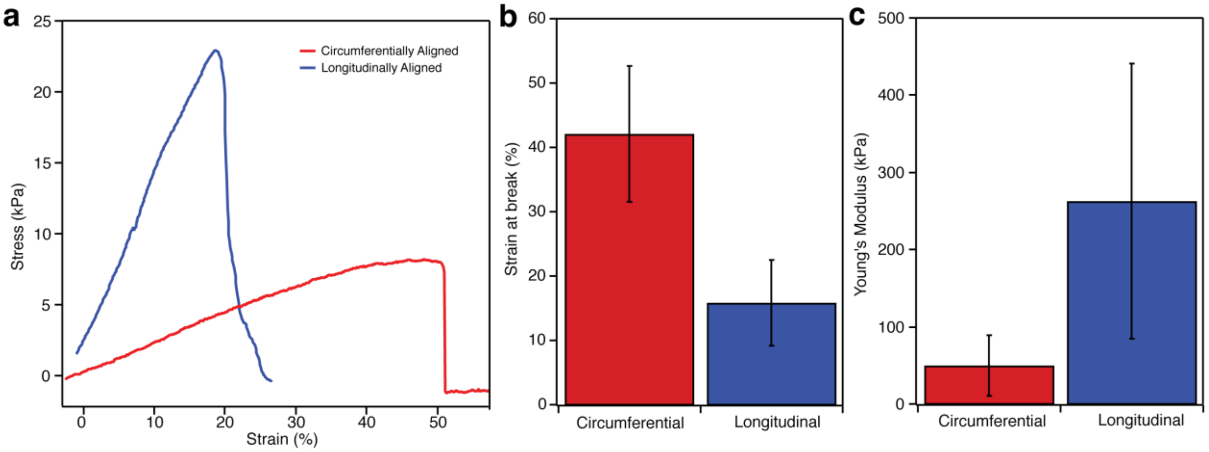

Supplementary Figure 14 | Tensile experiments of circumferentially (red) and longitudinally aligned (blue) hybrid tubes. (**a**) Representative examples of tensile tests. (**b**) Mean engineering strain at break (%) and (**c**) mean Young's modulus (kPa). Experiments show the circumferentially aligned hybrids (where the tensile force is applied perpendicular to nanofiber alignment) have higher strain at break than longitudinally aligned hybrids where tensile force is applied parallel to the direction of nanofiber alignment (42.1 ± 10.6 % vs 15.8 ± 6.7 %). Conversely, the circumferentially aligned samples show lower modulus (50.3 ± 39 kPa vs 263.3 ± 179 kPa). Data is represented as mean ± s.d. of 6–12 samples.

Supplementary Note 3 | Finite Element Analysis

1. Isotropic and anisotropic swelling of hydrogel

For an isotropic swelling hydrogel, the polymer network deforms homogeneously, and $\lambda = \lambda_x = \lambda_y = \lambda_z = \phi^{-1/3}$. Following the notation in the main text we can rewrite **Eq. 1** as

$$F(\phi,T) = \frac{1}{2} NkT\left(\frac{3}{\phi^{2/3}} - 3 + \ln\phi\right) + kT\frac{V_m}{\nu}\left[\left(\frac{1}{\phi} - 1\right)\ln(1-\phi) + \chi(1-\phi)\right] \quad (1)$$

When the free swelling gel is in equilibrium with the external solvent, its osmotic pressure Π must be zero, i.e.,

$$\Pi = -\frac{\partial F(\phi,T)}{\partial V} = \frac{\phi^2}{V_m}\frac{\partial F(\phi,T)}{\partial \phi} = 0 \quad (2)$$

where the number of monomers is assumed to be constant. Inserting **Supplementary Equation 1** in **Supplementary Equation 2** and substituting ϕ with λ^{-3} gives

$$\frac{\chi}{\lambda^3} = \frac{1}{2}\frac{N\nu}{V_m}\left(1 - 2\lambda^2\right) - \lambda^3 \ln\left(1 - \frac{1}{\lambda^3}\right) - 1 \quad (3)$$

For an anisotropic gel constrained along *z* direction, $\lambda = \lambda_x = \lambda_y = (\phi\lambda_z)^{-1/2}$. The equilibrium is reached when the stress in the free directions (i.e., *x* and *y* directions) equals to zero, which gives

$$\frac{\chi}{\lambda^2\lambda_z} = \frac{1}{2}\frac{N\nu}{V_m}\left(1 - 2\lambda^2\right) - \lambda^2\lambda_z \ln\left(1 - \frac{1}{\lambda^2\lambda_z}\right) - 1 \quad (4)$$

When $\lambda = \lambda_z$, the stretching ratios in all directions are equal, thus **Supplementary Equation 4** reduces to **Supplementary Equation 3**, indicating that the anisotropic gel reaches an isotropic swollen state. The corresponding χ given by **Supplementary Equation 4** at $\lambda = \lambda_z$ is denoted by χ_c in the following texts.

For an anisotropic gel constrained by rigid fibers oriented to the *z*-axis, λ_z is homogeneous if the fibers are assumed to be infinitely long, while λ_x and λ_y are inhomogeneous due to the constraint from the fibers. As such, it is challenging to achieve an analytical solution of the $(\lambda_x, \lambda_y, \lambda_z)$ distribution at equilibrium, and we therefore follow a finite element approach to numerically solve λ_x and λ_y with a fixed value of λ_z under periodic boundary conditions.

2. Finite element analysis

The finite element simulations are performed in the commercial software ABAQUS. The constitutive model of the hydrogel (**Eq. 1** in the main text) is implemented using the UHYPER subroutine. The hydrogel matrix is meshed with C3D8R elements. In all the simulations, the mesh density is validated in mesh convergence studies. The periodic boundary conditions are imposed as follows: Let S^+ and S^- be a pair of opposite boundaries defined by the lattice vector $\mathbf{l}$; for an arbitrary point located at $\mathbf{r}$ on S^+ and its duplicate point located at $\mathbf{r} - \mathbf{l}$ on S^-, their displacements $\mathbf{u}(\mathbf{r})$ and $\mathbf{u}(\mathbf{r} - \mathbf{l})$ must satisfy

$$\mathbf{u}(\mathbf{r}) - \mathbf{u}(\mathbf{r} - \mathbf{l}) = \mathbf{l}' - \mathbf{l} \tag{5}$$

where $\mathbf{l}'$ is the deformed lattice vector. Because $\mathbf{l}' - \mathbf{l}$ is the same for all pairs of duplicate points on S^+ and S^-, **Supplementary Equation 5** can be easily implemented using constraint equations in ABAQUS.

Consistent with the contraction experiments described in the main text, the dimensional change of a constrained gel is induced in the simulation when $\chi(T)$ varies. Specifically, the initial and final χ (denoted by $\chi(T_i)$ and $\chi(T_f)$, respectively) are defined by the given initial volume fraction and shrinkage for an isotropic shrinking gel. At χ_c, a constrained gel unit cell with a PA nanofiber embedded is in equilibrium and stress-free; when its χ varies from χ_c to $\chi(T_i)$ and $\chi(T_f)$, the constrained gel contracts correspondingly and we can then measure the contraction ratio from $\chi(T_i)$ to $\chi(T_f)$.

In the simulations, the pre-defined isotropic contraction of the swelling gel is 77%. The initial stretching ratio of the covalent polymer gel at room temperature is estimated to be 1.77. Using **Supplementary Equation 3**, we can calculate the corresponding $\chi(T_i)$ and $\chi(T_f)$ and use them for the anisotropic gel

contraction simulations. The parameter χ_c is calculated from **Supplementary Equation 4** using $\lambda = \lambda_z = 3.54$. The dimensionless quantity $N\nu / V_m$ is estimated as followed. Let N_{CL}, m_{CL}, M_{CL} denote the number, mass and molar mass of the crosslinkers respectively, $\rho_m \approx 10^6\ g\, m^{-3}$ be the density of the DEGMA/OEGMA polymer, and $\nu = 3\times10^{-29} m^3$ be the volume of a single water molecule. The weight percentage of the crosslinkers with respect to the weight of the polymer is $w_{CL} = 1\%$. The number of chains between the adjacent crosslinking junctions is given by $N = \frac{1}{2}(4N_{CL}) = 2N_A m_{CL} / M_{CL}$ assuming that 4 subchains are attached to each crosslinker. $V_m = m_m / \rho_m$ where m_m is the mass of the polymer. $M_{CL} = 154\, g\, mol^{-1}$. Therefore,

$$\frac{N\nu}{V_m} = \frac{2N_A m_{CL} \nu \rho_m}{M_{CL} m_m} = \frac{2N_A w_{CL} \nu \rho_m}{M_{CL}} \approx 2\times10^{-3} \quad \textbf{(6)}$$

It is noteworthy that the PA nanofibers are modeled as rigid rods in our simulations due to the remarkable difference between the moduli of the gel matrix and the PA nanofiber. Per **Eq. 1** in the main text, the initial shear modulus (when $\lambda_x = \lambda_y = \lambda_z = 1$) of the gel matrix is given by NkT/V_m. Assuming a Poisson ratio of 0.5, the Young's modulus of the gel matrix $E_{matrix} \sim 3NkT/V_m$. On the other hand, the Young's modulus of the PA nanofiber can be related to its persistence length l_p via $E_{fiber} \sim l_p kT/l$,[1, 2] where $l = \pi r^4/4$ is the geometrical moment of inertia of a cylindrical rod with a radius of *r*.[2] Thus,

$$E_{fiber}/E_{matrix} - \frac{4}{3}\pi\, l_p V_m / N r^4 \quad \textbf{(7)}$$

In our system, we assume $r = 5\ nm$, $l_p = 50\,\mu m$ based on persistence lengths in the micrometer range[3] previously determined for other PA nanofibers; by substituting **Supplementary Equation 6** in **Supplementary Equation 7** we have $E_{fiber}/E_{matrix} = 434$. As such, using a deformable fiber instead of a rigid one only results in a negligible change (<0.1%) of the contraction ratio in our simulations. Therefore, it is adequate to calculate the contraction of the polymer hybrid without considering the deformation of the PA nanofibers.

3. Estimating interfiber distances in the tubular hybrids

We used the dimensions of the mold (outer diameter = 4 mm, inner diameter = 3 mm, and length = 32 mm to calculate a tube volume of $V_{tube} = 175.23\, mm^3$) and the known mass concentration (10 mg mL^{-1}) of the PA solution filling the mold prior to gelation, to calculate the amount of PA per tube (1.75 mg). From atomistic simulations of similar PA nanofibers,[4] we obtained an approximate density of 0.778 mg mm^{-3} for the PA within the tubes. We assume a fiber diameter of 10 nm, which is consistent with data obtained from small angle x-ray scattering. From these parameters, we calculate a volume of $V_{fiber} = 2.249\ mm^3$ to be occupied by the PA nanofibers within the tube. Assuming the PA nanofibers to be arranged in a 2D square lattice as used in the finite element analysis, we can calculate the interfiber distance by solving the following equation for the width of the lattice, a:

$$\frac{\pi \times r^2}{a^2} = \frac{V_{fiber}}{V_{tube}} \tag{8}$$

Therefore, the interfiber distance can be estimated to be:

$$a = \sqrt{\frac{\pi \times r^2 V_{tube}}{V_{fiber}}} = 78\, nm \tag{9}$$

Supplementary Note 4 | Molecular Theory

Free energy model One PA polymer hybrid nanofiber is modeled as a cylinder of radius *R* that is end-tethered with N_p polymers chains covalently end-tethered to a cylindrical surface of area *A*(*R*). Each copolymer contains temperature-responsive ethylene glycol monomers (denoted as *A*) and hydrophobic monomers (denoted as *B*). Here, we present a description of the theory outlining its most important features. A complementary description of the theory for planar surfaces was previously described.[5] The total Helmholtz free energy of the system has the following contributions:

$$F = -TS_{conf} - TS_{mix} + E_{VdW} + F_{assoc}, \tag{10}$$

where *T* is the temperature; S_{conf} is the conformational entropy of the polymers chains; S_{mix} is the translational entropy of the water; E_{VdW} corresponds to the Van der Waals attraction between the monomers; F_{assoc} is the free energy of hydrogen bond formation, i.e., the enthalpic and entropic cost

associated with forming water-water and water-monomer hydrogen bonds. In the following a description of each of the terms of the free energy is presented.

The conformational entropy of the polymer chains [6], which per unit area, is given by

$$-\frac{S_{conf}}{k_B A(R)} = \sigma \sum_{\alpha} P(\alpha) \ln P(\alpha), \qquad \textbf{(11)}$$

here $\sigma = N_p / A(R)$ is the grafting density or surface coverage, i.e., the number of chains per unit area. $P(\alpha)$ corresponds to the probability to find a chain in conformation α. This probability distribution function (pdf) is essential in describing various thermodynamic and structural quantities related to the polymers. For instance, the average polymer number density, $\langle \rho_i(r) \rangle$, for monomers of type *i* at position *r* is given by

$$\langle \rho_i(z) \rangle = \frac{\sigma}{G(r)} \sum_{\alpha} P(\alpha) n_i(\alpha; r). \qquad \textbf{(12)}$$

Here $n_i(\alpha; r) dr$ is the number of polymer segments of monomer type *i* found within a volume element $[r, r+dr]$ that belong to polymer conformation α. The variable $n_i(\alpha; r) dr$ is input and dependent on the molecular architecture and chemistry of the polymer. The variable *r* is the radial coordinate in cylindrical coordinates. The system is assumed to be laterally homogeneous and only explicitly anisotropic in the radial cylindrical direction. The function $G(r) = A(r) / A(R) = r / R$ is the Jacobian determinant divided by the area of the cylindrical surface of the hybrid. It describes the change in volume as function of distance away from the nanofiber. The total polymer volume fraction is the sum of the volume fractions of the individual monomer of type A and B and is given by

$$\langle \phi(r) \rangle = \langle \phi_A(r) \rangle + \langle \phi_B(r) \rangle = \langle \rho_A(r) \rangle \upsilon_A + \langle \rho_B(r) \rangle \upsilon_B, \qquad \textbf{(13)}$$

where υ_A and υ_B is the volume of monomer of type A and B respectively.

The second term in the free energy of **Supplementary Equation 10** is the mixing entropy of the water molecules and it is given by

$$-\frac{S_{mix}}{k_B A(R)} = \int dr\ \rho_w(r)\left[\ln\left(\rho_w(r)\upsilon_w\right)-1\right], \tag{14}$$

here $\rho_w(r)$ is the number density of water and υ_w is the volume of water.

The third term in the free energy describes the attractive Van der Waals interaction energy between monomers,

$$\frac{\beta E_{VdW}}{A(R)} = -\frac{\chi_A}{2\upsilon_w}\int dr\ G(r)\langle\phi_A(r)\rangle^2 - \frac{\chi_B}{2\upsilon_w}\int dr\ G(r)\langle\phi_B(r)\rangle^2. \tag{15}$$

where χ_i is the interaction parameter that measures the strength of attraction between like-monomers, being either temperature responsive (A) or hydrophobic (B) monomers. In our calculations, we take $\chi_i = B_i / T$. Considering only the above Van der Waals interaction energy would result in a phase behavior and thermal response characteristic of UCST (upper critical solution temperature) systems. Hydrophobic monomers, which have a UCST phase behavior, are appropriately described by above interaction energy. However, PEG polymers have LCST behavior, which has its origins in hydrogen bond formation between PEG monomers and water molecules. Thus we include an additional term in the free energy, the fourth term in **Supplementary Equation 10**, which describes the free energy contribution of hydrogen bond formation between monomer and water and water-water molecules.[5, 7] This contribution is represented by:

$$\begin{aligned}
&\frac{\beta F_{assoc}}{A(R)} \\
&= \int dr\ G(r)2\langle\rho_A(r)\rangle\left[x_p(r)\ln x_p(r)+\left(1-x_p(r)\right)\ln\left(1-x_p(r)\right)-x_p(r)\beta\Delta F_p\right] \\
&+ \int dr\ G(r)2\rho_w(r)\left[x_w(r)\ln x_w(r)+\left(1-x_w(r)\right)\ln\left(1-x_w(r)\right)-x_w(r)\beta\Delta F_w\right] \\
&+ \int dr\ G(r)2\langle\rho_A(r)\rangle\left[1-x_w(r)-x_p(r)\frac{\langle\rho_A(r)\rangle}{\rho_w(r)}\right]\ln\left[1-x_w(r)-x_p(z)\frac{\langle\rho_A(r)\rangle}{\rho_w(r)}\right] \\
&- \int dr 2\rho_w(r)\left[x_w(r)+x_p(r)\frac{\langle\rho_A(r)\rangle}{\rho_w(r)}\right]\ln\frac{2\rho_w(r)\upsilon_w}{e}.
\end{aligned} \tag{16}$$

where $x_p(r)$ is the local fraction of polymer-water hydrogen bonds for A monomers and $x_w(r)$ is the local fraction of water-water hydrogen bonds. Only hydrogen bonds formation between the A (PEG)

monomers and water molecules and between water-water molecules are considered. The B monomers do not form hydrogen bonds with water. In **Supplementary Equation 17**, the free energy contribution $\beta\Delta F_i = \beta\Delta E_i - \Delta S_i / k_B$ is the sum of the energetic gain (ΔE_i) of forming one hydrogen bond and the corresponding entropic loss (ΔS_i) of forming the hydrogen bond for either *i* = *p* (polymer-water) or *i* = *w* (water-water) hydrogen bond pair. They have the following values:

$$\beta\Delta F_p = \frac{2000\,K}{T} - 3.35 \text{ and } \beta\Delta F_w = \frac{1800\,K}{T} - 2.25. \quad \textbf{(17)}$$

These values, obtained from experiments and simulation results, are specific for PEG and water [7]. A derivation of this free energy contribution was described previously [5, 7].

The repulsive interactions in the theory are modeled as excluded volume interactions. The intrachain interactions are explicitly considered during generation of the polymer conformations. The intermolecular excluded volume interactions are accounted for by assuming that the system is incompressible at every position,

$$\langle \phi_p(r) \rangle + \rho_w(z)\upsilon_w = 1. \quad \textbf{(18)}$$

These volume constraints are enforced through introduction of the Lagrange multipliers $\pi(r)$.

The free energy expression is minimized with respect to $P(\alpha)$, $\rho_w(r)$, $x_w(r)$, and $x_p(r)$, under the constraint of incompressibility. Minimization with respect to $P(\alpha)$ yields,

$$\begin{aligned} P(\alpha) = \frac{1}{q}\exp&\left[-\beta\int dr\,\pi(r)n_A(\alpha;r)\upsilon_A + \frac{\chi_A}{\upsilon_w}\int dr\,\langle\phi_A(r)\rangle n_A(\alpha;r)\upsilon_A - 2\int dr n_A(\alpha;r)\ln\left(1-x_p(r)\right)\right] \\ &\times\exp\left[-\beta\int dr\,\pi(r)n_B(\alpha;r)\upsilon_B + \frac{\chi_B}{\upsilon_w}\int dr\,\langle\phi_B(r)\rangle n_B(\alpha;r)\upsilon_B\right], \end{aligned} \quad \textbf{(19)}$$

where *q* ensures normalization of $P(\alpha)$ and $\pi(r)$ is the Lagrange multiplier or lateral pressure field that enforces the incompressibility constraint.

Minimizing with respect to the water number density or volume fraction gives

$$\phi_w(r) = \exp\left[-\beta\pi(r)\upsilon_w - 2\ln\left(1-x_w(r)\right) - 2\ln\left(1-x_w(r)-x_p(r)\frac{\langle\rho_A(r)\rangle}{\rho_w(r)}\right)\right]. \quad (\mathbf{20})$$

Finally, minimizing the free energy expression with respect to the local fraction of polymer-water $x_p(r)$ and water-water hydrogen bonds $x_w(r)$ gives

$$x_p(r) = 2\exp\left(\beta\Delta F_p\right)\phi_w(r)\left(1-x_p(r)\right)\left(1-x_w(r)-x_p(r)\frac{\langle\rho_A(r)\rangle}{\rho_w(r)}\right), \quad (\mathbf{21})$$

and

$$x_w(r) = 2\exp\left(\beta\Delta F_w\right)\phi_w(r)\left(1-x_w(r)\right)\left(1-x_w(r)-x_p(r)\frac{\langle\rho_A(r)\rangle}{\rho_w(r)}\right). \quad (\mathbf{22})$$

The unknowns in **Supplementary Equation 13**, and **Supplementary Equation 19** through **Supplementary Equation 22** are the Lagrange multipliers or lateral pressures, $\pi(r)$, the local fraction of polymer-water $x_p(r)$ and water-water hydrogen bonds $x_w(r)$. Once solutions for these variables are found all equilibrium thermodynamic and structural properties of the system are known. Solutions of these variables can be obtained numerically. This is accomplished by substituting expressions of the polymer and water volume fractions into the incompressibility constraint **Supplementary Equation 18** and the 'chemical reaction equations' of **Supplementary Equation 21** and **Supplementary Equation 22**, which results in a set of non-linear integro differential equations. By discretizing space these differential equations can be converted into a set of coupled non-linear algebraic equations that can be solved with standard numerical techniques [8]. Details on the discretization procedure and numerical methods can be found in the literature [5, 9].

Finally, we employ a three-state RIS model to generate the chain conformations [10]. In this model, each bond has three different isoenergetic states. The conformations are generated by a simple sampling method and all are self-avoiding and cannot penetrate into the nanofiber. We generated a set of independent conformations for each different molecular weight. The same set of conformations was used in all calculations for a given molecular weight. Each segment of the polymers had a length $l_{seg} = 0.30\,nm$, and a volume $\upsilon_A = \upsilon_B = 0.065\ nm^3$ which corresponds to the partial molar volume of PEG in water. The volume of a water molecule was $\upsilon_w = 0.03\,nm^3$. The interaction parameter for the ethylene glycol monomers is fixed at $\chi = 100/T$. The hydrophobic monomer is not allowed to form

hydrogen bond between monomer and water. The discretization thickness was $\delta = 0.35 - 0.5\ nm$, which is sufficient to obtain accurate solutions for the given set of equations.

Thermal Response of PEG. To understand in more detail the role of hydrogen bonding on the thermal response of the PEG-like cylindrical brush we present in **Supplementary Figure 15** the average fraction of water-water hydrogen bonds and polymer-water hydrogen bond as function of temperature for a PA nanofiber end-tethered with PEG polymers. Adjacent to that the polymer volume fraction as function of distance away of the nanofiber is shown. The number of water-water and polymer-water hydrogen bond is like the polymer volume fraction position dependent. Therefore, in order to be able to compare different temperatures we introduce the spatial average fraction of PEG-water hydrogen bonds, $\langle x_p \rangle = \int dr\, G(r) x_p(r) \langle \rho_A(r) \rangle / \int dr\, G(r) \langle \rho_A(r) \rangle$ and the average fraction of water-water hydrogen bonds, $\langle x_w \rangle = \int dr\, G(r) x_w(r) \rho_w(r) / \int dr\, G(r) \rho_w(r)$. The integration is restricted to the region where there are polymers, otherwise if the integration is extended to infinity $\langle x_w \rangle$ would become equal to the average fraction of water-water hydrogen bonds of pure water. Observe that the formation of hydrogen bonds is always accompanied by a gain in free energy, that is, both $-\Delta F_p$ and $-\Delta F_w$ are negative (see **Supplementary Equation 18**). However, the free energy gain to form water-water hydrogen bonds is larger than the free energy gain to form polymer –water hydrogen bonds $\left(\Delta F_p < \Delta F_w\right)$. Consequently, when temperature increases the free energy gain to form polymer-water hydrogen bonds decreases quicker than the decreases in free energy gain to form water-water hydrogen bonds. This effect is demonstrated in **Supplementary Figure 15**.

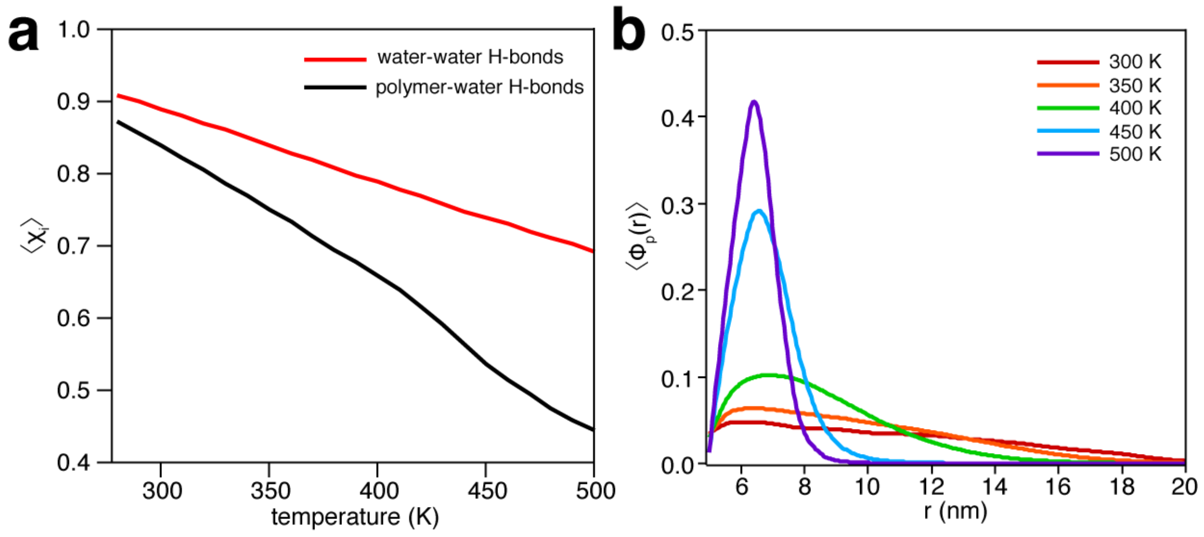

Supplementary Figure 15 | (**a**) Spatial average fraction of polymer-water hydrogen bonds and water-water hydrogen bonds as function of temperature for the PA nanofiber end-tethered with PEG. (**b**) The polymer volume fraction versus radial distance away from the PA nanofiber for different temperatures. Radius of the nanofiber is 5 nm, N_p=200, and the surface coverage is 0.07 chains nm^{-2}.

Hence water increasingly prefers to have hydrogen bonds with other water molecules instead of having hydrogen bonds with polymer segments. This effectively leads to a decrease in the solvent quality. With increasing temperature, the solvent quality decreases and is able to overcome the conformational entropy of the tethered polymer, which favors extended conformations. Therefore, the polymer layer shrinks, and at elevated temperatures the layer eventually collapses (**Supplementary Figure 15b**).

Thermal Response of PEG-like copolymer. The LCST of pure PEG polymers is above 400K.[7] Therefore, we considered a very large and experimentally unrealistic temperature range. By introducing hydrophobic monomers into the copolymer, a drastic reduction of the transition temperature can be accomplished as displayed in **Fig. 5c**. Whereas a PEG polymer has a transition temperature of around 425 K, a linear alternating copolymer $(EG_2\text{-a-HB})_{66}\text{-}EG_2$ (blue curve of **Fig. 5c**) transitions around 325 K from a swollen, extended state to a collapsed state. These predictions are consistent with the experimental observations by Lutz *et al.* showing that the fraction of hydrophobic monomers controls the LCST behavior for PEG copolymers similar to one considered in this paper [11].

The drastic reduction is observed because the hydrophobic monomers are not allowed to form hydrogen bonds between monomer and water. Consequently, a water-hydrophobic monomer contact is energetically very unfavorable. There is no energetic gain, unlike forming hydrogen bonds with other water molecules and with ethylene glycol monomers. This effectively causes the copolymer to experience very poor solvent quality, and the brushes collapse at a much lower temperature than the pure PEG polymer.

Note, the effect of varying the interaction parameter for the hydrophobic monomers is very small because the effective repulsions between the hydrophobic monomer and water molecules are mostly mediated indirectly by the hydrogen bond formation between water molecules, and not by the effective Van der Waals interaction. The height of polymer layer for the end-tethered copolymers, presented in **Fig. 5c**, has only a very limited dependence on χ_B. The interaction parameter was arbitrarily set to $\chi_B = 0.15/T$.

Thermal Response effect of surface density and molecular weight. In **Supplementary Figure 16** we explore the effect of surface coverage and molecular weight or degree of polymerization on the thermal response of end-tethered PEG. We observe that with increasing surface coverage the height of the swollen state increases. This is due to the increased excluded volume interaction resulting from the increased polymer density near the nanofiber surface. Likewise, due to the increase in the number of monomers the collapsed state is also larger. A similar but much more pronounced trend can be observed with increases in chain length. Longer chains can stretch further away from the surface and form more energetically favorable hydrogen bond pairs with water molecules. Consequently, longer chains form a relatively more extended state. Effects of the variation of the density of initiators on the structure of the polymers are obtained via molecular modeling (shown in **Supplementary Figure 16a**). We expect some increase in actuation with increased grafting above the experimental grafting density of 0.07 chains nm^{-2}; however, at high grafting densities the changes are minimal due to increased sterics preventing polymer mobility close to the nanofiber surface. This is corroborated experimentally in **Supplementary Figure 17,** where increasing the grafting density by increasing the mol% of initiator in the supramolecular coassembly. The overall degree of contraction remains similar across three cases; however, the kinetics of the contraction appear to change. These kinetics will be explored in future work.

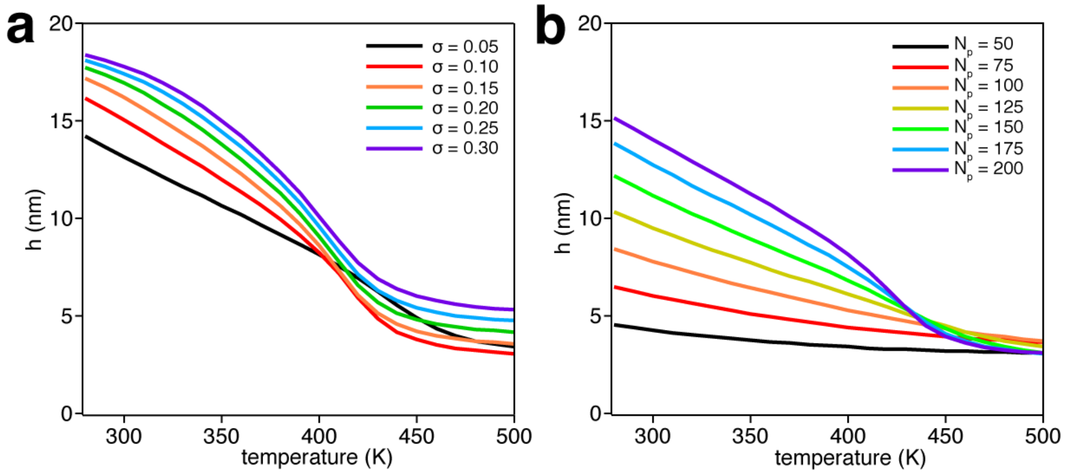

Supplementary Figure 16 | (**a**) Height of end-tethered PEG polymers as function of temperature for different grafting densities for fixed number of segments (N_p=200). (**b**) Height of end-tethered PEG polymers as function of temperature for different polymer chain lengths for fixed surface coverage of 0.07 chains nm^{-2}. Radius of the nanofiber is 5 nm.

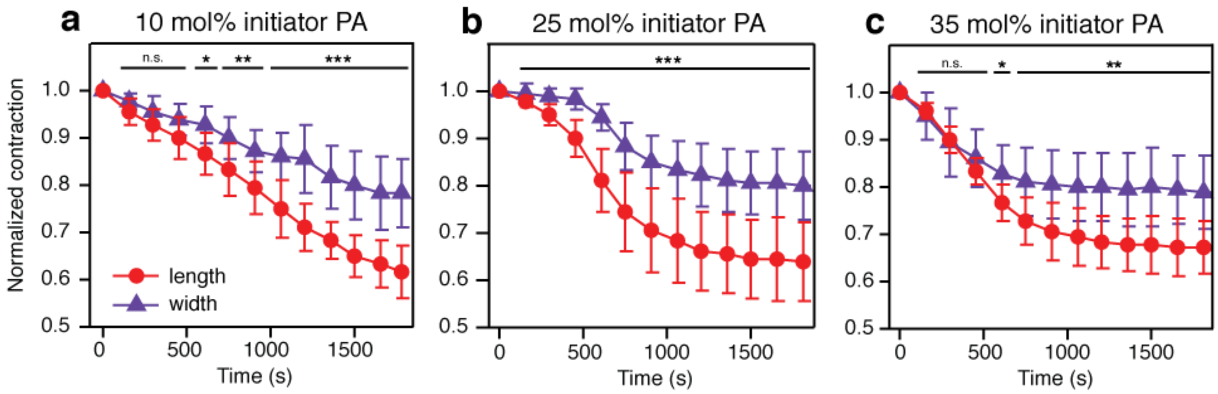

Supplemental Figure 17 | The effects of modifying grafting density on actuation at (**a**) 10 mol% **PA1**, (**b**) 25 mol% **PA1**, (**c**) 35 mol% **PA1**. Statistical analysis was performed using an unpaired two samples Student´s t-test; * $p<0.05$, ** $p<0.01$, *** $p<0.001$; (Data are presented as mean ± s.d., n = 3).

Supplementary Note 5 | Effect of crosslinking on material opacity and thermoresponse. The continuum model shows that an important parameter for the degree of anisotropy is related to the degree of crosslinking within the gel as well as the response of the gel to temperature variations via $\chi(T)$, which is provided by the molecular theory in the absence of crosslinking (**Supplementary Figure 15**). However, the gel degree of collapse is restricted by the constraint imposed by crosslinking, which are similar to the constraints imposed by grafting chains on a surface. In this case heterogeneities develop, as evident in the lateral pressure as a function of area per grafted chain with increasing temperature (**Supplementary Figure 18**). The collapse at high temperature leads to a coexistence of two-phase composition due to the constraints in the grafted case vertical and lateral collapsed state. These constraints are similar to those in a crosslinked gel, which always have polydispersity in the number of monomers per crosslink causing regions with different monomer densities, providing an opaque color to the collapsed gels. We note that the number of monomers per crosslink is directly related to the number density of accessible chains for polymer growth from the fiber surface.

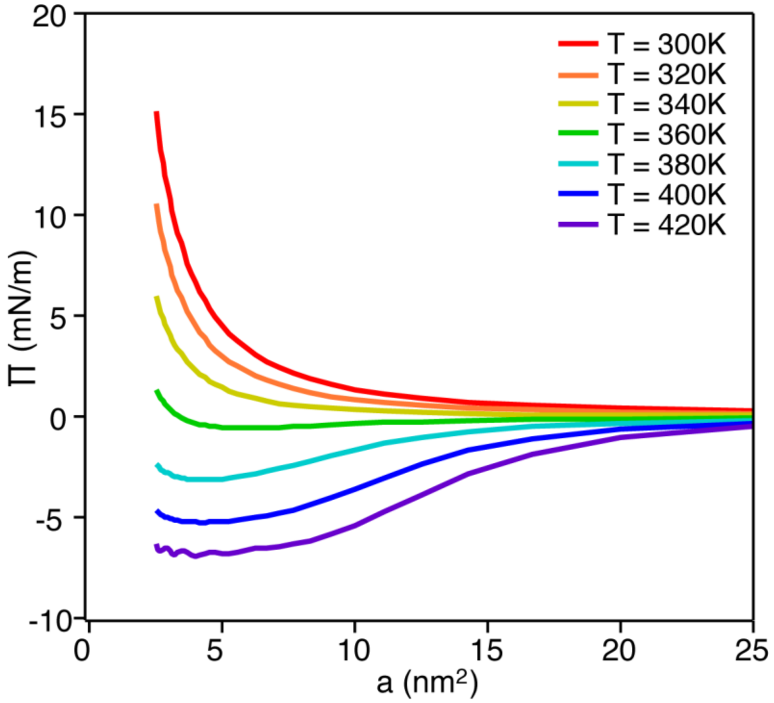

Supplementary Figure 18 | Pressure-area isotherms, lateral pressure as a function of the area per molecule, $a = A(R)/N_p$. The polymer is a linear alternating copolymer of PEG and hydrophobic monomers, with a ratio of 4:1. The number of monomers is $N_p = 150$, and the radius of the nanofiber is 5 nm.

Supplementary Note 6 | Bending of 3D printed hybrid sheets. As shown in **Figure 6** of the main text, the aligned hybrid sheets bend unidirectionally, towards the printing substrate and perpendicular to the aligned fiber axis, upon increase in the surrounding temperature above the transition temperature of the covalent polymer. This bending is highly reversible and repeatable over multiple cycles. Similar bending and even rolling behavior of polymer sheets has been observed in the case of bilayers.[12, 13] However, these materials typically bend due to the mismatch in moduli or expansion coefficients between the two components of the bilayer. In contrast, the hybrid sheets presented here have a homogeneous dispersion of supramolecular nanofibers within the polymer matrix, as shown in the cross-sectional confocal microscopy image (**Supplementary Figure 19**). In polarized microscopy images, we find increased birefringence of the layers closest to the substrate, indicating stronger alignment, compared to the upper layers of the sheets (**Supplementary Figure 20**). SEM imaging also suggests that there is better alignment on the bottom surface of the sheets than on the top surface (**Supplementary Figure 21**). We hypothesize that the first layer may have a higher drag shear force during extrusion onto a hard glass substrate than the drag shear force experienced by the upper layers when extruded onto the soft hydrogel surface of the initial previous layer. The differences in alignment between layers may be responsible for the preferential direction of bending in the hybrid sheets, where the more aligned bottom layer contracts more along the direction perpendicular to fiber alignment than the top layers, resulting in a net bending motion towards the bottom of the gel. Interestingly, hybrid sheets that have a cross-hatch or square spiral pattern and do not feature net alignment, show a buckling behavior instead

of a bending motion (**Figure 6**, **Supplementary Figure 22**). This indicates that the stresses generated by the transition of the thermo-responsive polymer cannot be released in a directed motion as is the case for sheets having a net alignment. We anticipate this feature to be useful in designing more complex macroscopic responses of our material by using pre-programmed patterns of the 3D printed material, inspired by the work of the Lewis group.[14]

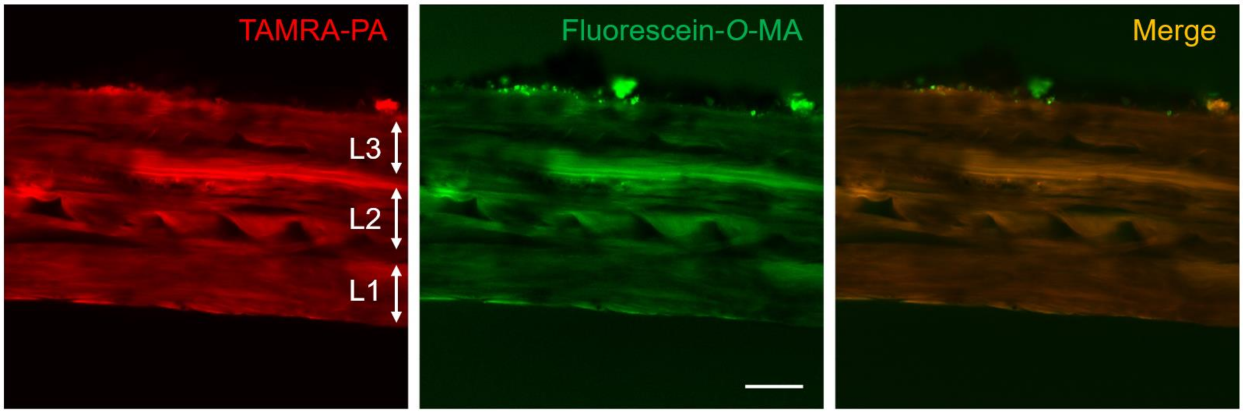

Supplementary Figure 19 | Confocal microscopy of the cross-section of a 3D printed three layer aligned sheet. L1 represents the first layer printed on the glass substrate and L2 and L3 are the subsequent printed layers. Scale bar are 100 μm.

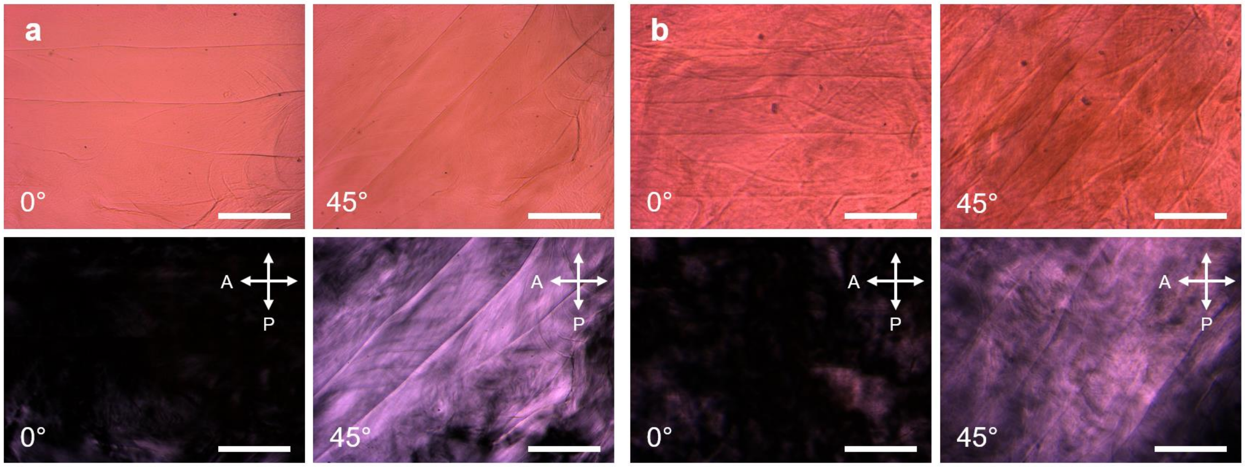

Supplementary Figure 20 | Optical microscopy of (**a**) one layer and (**b**) three layer aligned 3D printed sheets. When the sample is aligned 45° to the polarizer, a more uniform and stronger birefringence is observed for one layer sheets than for three layer sheets. Scale bars are 400 μm.

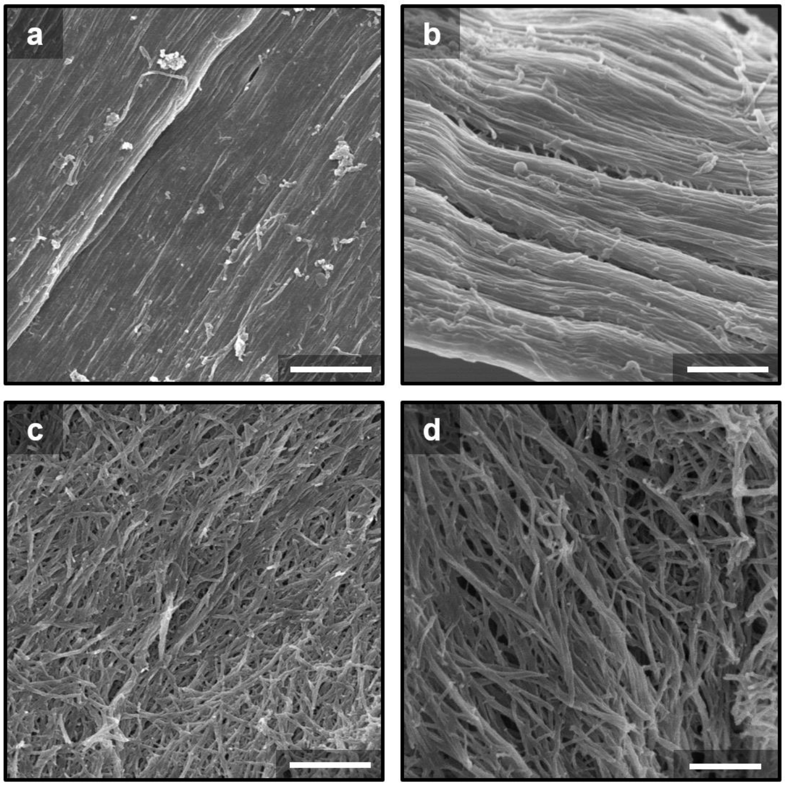

Supplementary Figure 21 | Representative SEM images from the bottom (**a**, **b**) and top (**c**, **d**) surfaces of a 3D printed three layer aligned sheet. Scale bars are 1 µm in (**a, b, c**) and 500 nm in (**d**).

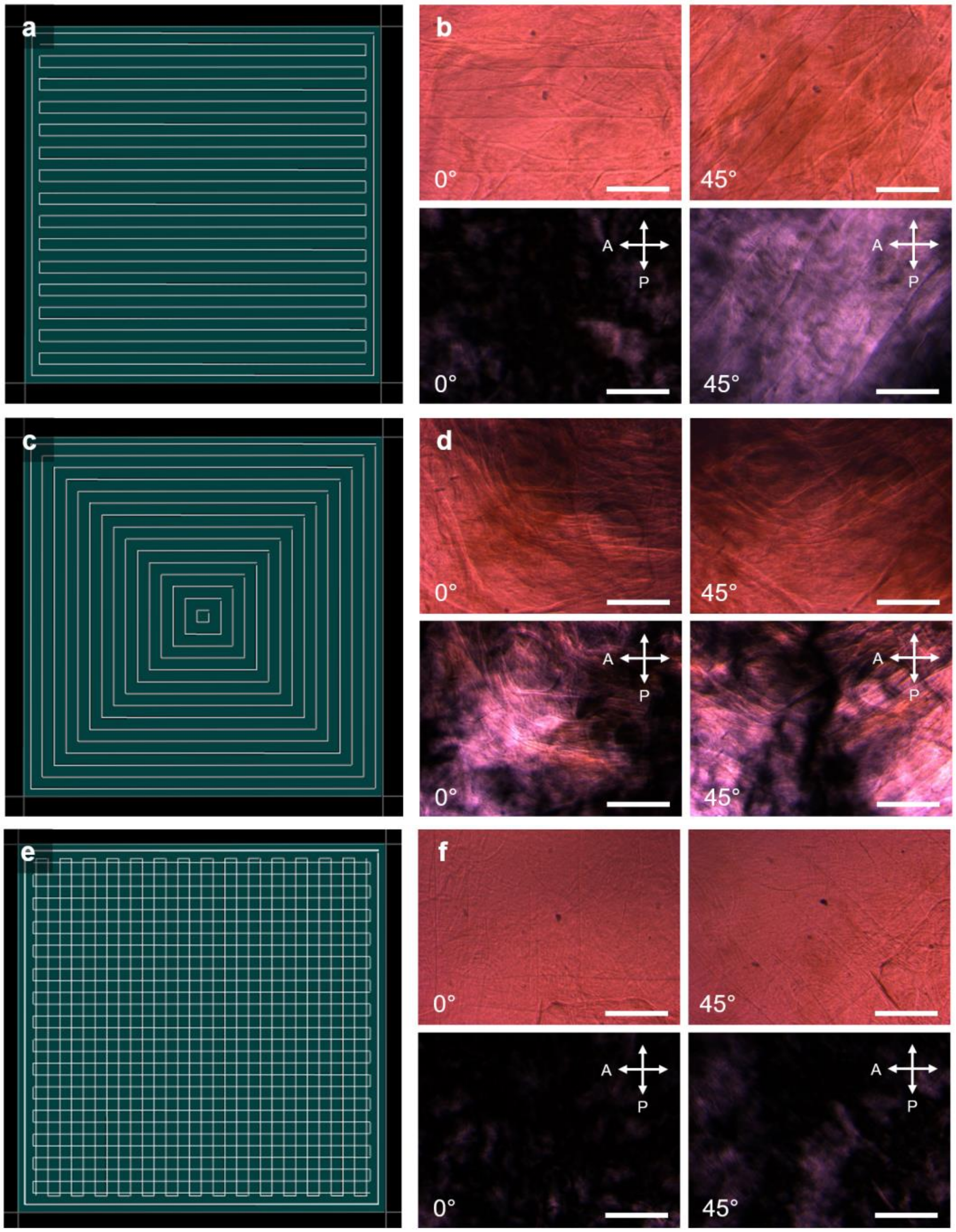

Supplementary Figure 22 | 3D printed PA-polymer hydrogel sheets with varying print paths. (**a**) Print path for a uniaxially aligned three-layer sheet. (**b**) Optical microscopy of an aligned three-layer sheet, demonstrating strong birefringence when the sample is rotated 45° to the polarizer. (**c**) Print path for a square spiral three-layer sheet. (**d**) Optical microscopy on a corner of a square spiral three-layer sheet, showing bright birefringence at the point of the corner when the sample is at 0°, but a dark region at the corner point when the sample is rotated 45° to the polarizer. At the corner point, the fibers bend at ~45° to connect one edge of the square to the adjacent edge. (**e**) Print path for a cross-hatch four-layer square sheet. (**f**) Optical microscopy of a cross-hatch four-layer sheet, with birefringence extinguished at both 0° and 45° because the layers are oriented 90° to each other. Scale bars are 400 μm.

Supplementary References:

1. Landau LD, LIfshitz EM. Statistical physics, part 1. In: *Statistical physics, part 1*. 3 edn. Butterworth-Heinemann (1980).

2. Landau LD, Lifshitz EM, Pitaevskii LP, Kosevich AM. Theory of elasticity. In: *Theory of elasticity*. Butterworth-Heinemann (1986).

3. Hung AM, Stupp SI. Understanding factors affecting alignment of self-assembling nanofibers patterned by sonication-assisted solution embossing. *Langmuir* **25**, 7084-7089 (2009).

4. Lee JB, Hong J, Bonner DK, Poon Z, Hammond PT. Self-assembled rna interference microsponges for efficient sirna delivery. *Nat. Mater.* **11**, 316-322 (2012).

5. Ren C-l, Nap R, Szleifer I. The role of hydrogen bonding in tethered polymer layers. *J. Phys. Chem. B.* **112**, 16238-16248 (2008).

6. Szleifer I, Carignano MA. Tethered polymer layers. In: *Advances in chemical physics*. John Wiley & Sons, Inc. (1996).

7. Dormidontova EE. Role of competitive peo— water and water— water hydrogen bonding in aqueous solution peo behavior. *Macromolecules* **35**, 987-1001 (2002).

8. Hindmarsh AC*, et al.* Sundials: Suite of nonlinear and differential/algebraic equation solvers. *ACM Transactions on Mathematical Software (TOMS)* **31**, 363-396 (2005).

9. Nap R, Gong P, Szleifer I. Weak polyelectrolytes tethered to surfaces: Effect of geometry, acid–base equilibrium and electrical permittivity. *J. Polym. Sci. B.* **44**, 2638-2662 (2006).

10. Flory PJ. *Statistical mechanics of chain molecules*. Interscience Publishers, Inc. (1969).

11. Lutz J-F, Hoth A. Preparation of ideal peg analogues with a tunable thermosensitivity by controlled radical copolymerization of 2-(2-methoxyethoxy) ethyl methacrylate and oligo (ethylene glycol) methacrylate. *Macromolecules* **39**, 893-896 (2006).

12. Hu Z, Zhang X, Li Y. Synthesis and application of modulated polymer gels. *Science* **269**, 525 (1995).

13. Ionov L. Hydrogel-based actuators: Possibilities and limitations. *Mater. Today* **17**, 494-503 (2014).

14. Gladman SA, Matsumoto EA, Nuzzo RG, Mahadevan L, Lewis JA. Biomimetic 4d printing. *Nat. Mater.* **15**, 413-418 (2016).
